# Application of adipose-derived stem cells in photoaging: basic science and literature review

**DOI:** 10.1186/s13287-020-01994-z

**Published:** 2020-11-23

**Authors:** Shidie Chen, Zhigang He, Jinghong Xu

**Affiliations:** Department of Plastic Surgery, The First Affiliated Hospital, School of Medicine, Zhejiang University, No. 79 Qingchun Road, Hangzhou, 310003 China

**Keywords:** Adipose-derived stem cell, Photoaging, Exosome, Conditioned medium, Reactive oxygen species, Aging, Secretome, Skin aging

## Abstract

Photoaging is mainly induced by continuous exposure to sun light, causing multiple unwanted skin characters and accelerating skin aging. Adipose-derived stem cells(ADSCs) are promising in supporting skin repair because of their significant antioxidant capacity and strong proliferation, differentiation, and migration ability, as well as their enriched secretome containing various growth factors and cytokines. The identification of the mechanisms by which ADSCs perform these functions for photoaging has great potential to explore therapeutic applications and combat skin aging. We also review the basic mechanisms of UV-induced skin aging and recent improvement in pre-clinical applications of ADSCs associated with photoaging. Results showed that ADSCs are potential to address photoaging problem and might treat skin cancer. Compared with ADSCs alone, the secretome-based approaches and different preconditionings of ADSCs are more promising to overcome the current limitations and enhance the anti-photoaging capacity.

## Introduction

The skin is our largest organ by weight and extent. It not only protects us from environment factors, but also synthesizes, processes, and metabolizes structural biomolecules such as lipid, protein, and glycan [1]. As a multifaceted organ, the skin also has sensory function and exerts pivotal role in esthetic appearance.

Skin aging is a culmination of intrinsic and extrinsic elements, which result in decreased structural integrity and disruption of normal physiological function. Extrinsic factors such as solar radiation, cigarette [2], or other pollution factors could induce skin aging. Among them, exposure to UV (long wavelength ultraviolet radiations (UVA) and medium wavelength ultraviolet radiations (UVB) exposure) radiation (UVR) is the major source of extrinsic skin aging, which is also known as photoaging.

Photoaging accounts for nearly 80% of facial aging [3]. It is characterized by fine wrinkles, dryness, laxity, rough texture, decreased elasticity, impaired wound healing, and benign and malignant growths [4–7]. It also exaggerates or accelerates the destruction of physiologic structure and the loss of various protective capacities, remaining an unsolved problem worldwide. In daily life, the condition of the skin is an important element used to estimate people’s age and health [8]. With the development of modern society and increasing life expectancy, maintaining a youthful and vigorous appearance is highly desired, which has facilitated the dramatic growth of the cosmeceutical industry.

Plant extracts [9], antioxidants [10], growth factors and cytokines [11], and stem cells [12] can be used to treat photoaging. Recently, stem cell therapy has attracted great attention because it can improve the regeneration ability of various tissues [12–14]. It is reported that stem cells and their derivatives are able to ameliorate skin conditions to some extent [15]. People are quite interested in the application of adipose-derived stem cells (ADSCs) in fields of dermatological and esthetic medicine, because they can be isolated and expanded easily and have clear multi-lineage differentiation [16–18]. Furthermore, it is reported that ADSCs can synthesize and secrete a lot of biologically active substances, mainly including antioxidants and cytokines that can be extracted and stored safely for a long time [19]. There are several reports in the past illustrated that ADSCs and their secretome can treat photoaging both in vivo and in vitro [20–22].

ADSCs improve conditions of photoaged skin, but their anti-photoaging mechanism is still elusive. The mechanism of ADSCs contributing to inflammation, wound healing, cancer has already been reviewed [23–25]. ADSCs against photoaging may share same molecular interactions and pathways with them because photoaging is related to inflammation, is similar to skin wound progress [26] and may result in skin cancer. This article reviews the mechanisms by which skin aging prevails following exposure to UV radiation as well as the recent research developments on the anti-photoaging effects of ADSCs and ADSC secretome. An overview of established and emerging treatment capacity of ADSCs and ADSC secretome, which have been proven or at least have demonstrated potential to inhibit or recupe- rate the unwanted clinical manifestations of photoaging, will be explored. Current research has come up with many new fascinating approaches to modify ADSCs or combine them with other materials to improve their treating ability. These novel ideas will be rendered in the manuscript(Table 1).

**Table 1:** Different preconditionings of ADSCs and their functions

Precondition	Capacities compared with ADSCs alone or untreated	Reference
CO_2_ laser combination	Further promote the activation of dermal fibroblast	[21]
GARP silencing	Increase their activation of TGF- *β* which augment the levels of mtROS	[30]
Nrf2 overexpressing	Promoted granulation tissue formation, angiogenesis, increased the expression of growth factor and decreased proteins related to inflammation and oxidation	[31]
HIF1 *α* overexpressing	Decrease oxidative stress and DNA damage	[32]
H_2_O_2_ treated	Reduce apoptosis, more skin flap survival area	[33]
VEGF overexpressing	Inhibit senescence by downregulating SA- *β*-Gal, recover UV-induced skin injury	[34]
Combined with nanofat	Ablate wrinkles	[35]
Transfected with miR-146a	Angiogenic and anti-inflammatory abilities	[36]
Combined with HA gel	Ablate wrinkles	[37]
Seeded onto collagen scaffolds	Increase dermal thickness and increase ECM	[38]
Combined with fat graft	Ablate wrinkles, promote collagen synthesis and neovascularization	[39]
LLL preconditioning	Increase growth factors secretion, increase dermal thickness	[40]
Engineered to express IFN- *β* and combined with cisplatin	Migrate to tumor sites and inhibit the growth of melanoma	[41]

## Molecular, cellular, and histological alternation of skin in photoaging

**Fig. 1:**
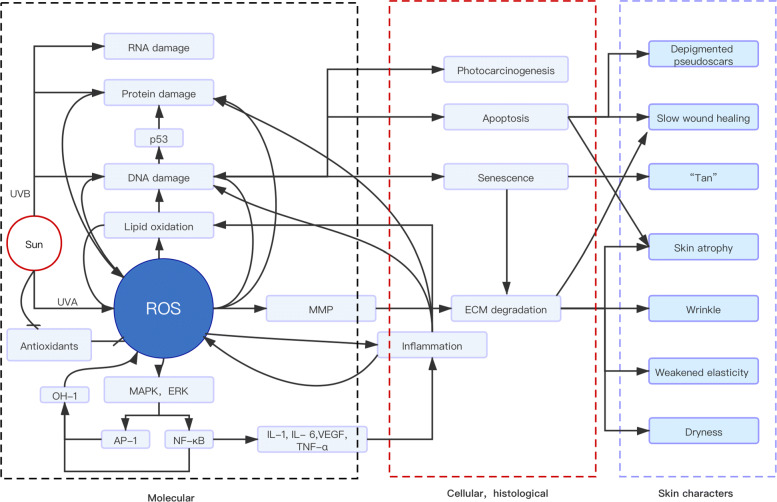
UV-induced skin aging model

The aged condition of photoaged skin is induced by molecular, cellular, and histological alternation and damage to the skin structure. UVR accelerates skin aging by causing direct and indirect damage to multiple skin structures. The simplified diagram of the general model of UV-induced skin aging is shown in Fig. 1.

### Direct damage induced by UVB

Direct damage is mainly caused by UVB. A considerate part of UVB is absorbed in the stratum corneum, and the rest part of UVB is absorbed in epidermal cells [27], inducing biological alternation in DNA, RNA, protein. DNA alternation is the most crucial one because accumulations of DNA damage can cause cell senescence and apoptosis. What is more, DNA alternation can damage the apoptotic capacity of skin cells and increase the possibility of malignancies [28].

### Reactive oxygen species (ROS)-related indirect damage

UVR can also cause physiological damage and accelerate skin aging indirectly via endogenous or exogenous photosensitizers that absorb solar radiations. The progress generates free radicals and ROS that induce skin inflammation. The inflammation progress can produce ROS by phagocytic cells and polynuclear lymphocytes [29]. Besides, inflammatory progress and ROS induce oxidative damage to DNA, cellular proteins, lipids, and carbohydrates [42], which in turn leads to more ROS production, resulting in a negative feedback loop [43]. Lipids are important targets of ROS and ROS can react with them in the cellular membrane to produce more reactive oxygen intermediates [44]. In addition, damage to the membrane lipids can induce structural damage in cell, cellular components leakage and eventually cell death [42].

Besides, ROS can cause indirect damage to DNA by oxidation products. For example, singlet oxygen, as the product of ROS, can turn guanine into 8-oxoguanine [45]. Low level of ROS can lead to mutation, medium levels can induce cell cycle arrest, and high levels can result in apoptosis and cell death [46]. UVR can also accelerate telomere shortening and lead to activation of p53. P53 is a tumor suppressor protein that can induce cell cycle arrest and apoptosis [47]. The apoptosis of stem cells in the basal layer induced by UVR is suggested to cause epidermal atrophy, slow wound healing, and depigmented pseudoscars [48]. On the other hand, some studies illustrated that UV-induced cell apoptosis exerts a protective function in UV injury [49–51]. After acute DNA damage, fibroblasts are more likely to undergo senescence rather than apoptosis and the dermis of photoaged skin does contain senescent fibroblasts that express senescence-associated *β* -galactosidase(SA- *β*- Gal) positivity [52]. Senescence reduces cell metabolic activity. Thus, the synthesis of elastic fibers and collagen fibers in the dermis is reduced. It finally results in weakened elasticity, and wrinkles of the skin [6, 53]. The greater melanin produced by senescent melanocytes could be related to the permanent “tan” noted in photoaged skin of people with darker complexions [54]. However, the molecular details associated with freckling, lentigines, and other pigment alternations of photoaged skin remain elusive [48].

Nevertheless, there are antioxidants in our body to maintain the oxygen homeostasis. Glutathione peroxidase (GPx) is the most essential antioxidant enzymes that remove free radicals. GPx can facilitate a reaction with the thiol-group of glutathione to eliminate singlet oxygen, hydrogen peroxide, and other peroxides [55]. Superoxide dismutase (SOD) and catalase can also remove superoxide radicals or hydrogen peroxide. However, the photoaged skin is reported to have reduced levels of natural enzymatic and non-enzymatic antioxidants [56], together with increased neutrophil infiltration and inflammation [57].

### Extracellular matrix(ECM) degradation

Lots of damage happens in the connective tissue, which is also known as the dermal ECM. Collagen, elastin, and glycosaminoglycans (GAGs) are the most critical and abundant substance of the dermal ECM. Features of photoaged skin include the accumulation of abnormal elastin fibers and GAGs, as well as collagen damage and reduction [58]. UVR-induced ROS stimulates the matrix-degenerating metalloproteases (MMPs) synthesis [42] that can induce the degradation of ECM. Alternatively, ROS can damage ECM indirectly by the oxidation products of DNA, lipid, and protein. Subsequently, several intracellular kinases such as mitogen-activated protein kinase (MAPK) and extracellular regulated protein kinases (ERK) will become activated. Ultimately, transcription factor complexes activator protein 1 (AP-1) and nuclear factor-kappaB (NF- *κ*B) will be produced and activate MMP transcription. Therefore, ROS can increase collagen degradation and aberrant elastin accumulation by altering gene expression pathways. Besides, heme oxygenase-1 (HO-1) can be induced by activated AP-1 and NF- *κ*B [59]. It can elevate free irons concentration and promote further ROS production through Fenton reaction [60]. Activated NF- *κ*B in fibroblasts can induce the transcription of proinflammatory cytokines interleukin (IL)-1, IL-6, vascular endothelial growth factor (VEGF), and tumor necrosis factor- *α* (TNF- *α*) [28], thus stimulating the inflammatory cells infiltration. These qualitative and quantitative alternations of ECM eventually lead to decreased tensile strength and recoil capacity, as well as wrinkle formation, dryness, wound healing damage, and an increase in brittleness. [61].

## ADSCs mechanisms in photoaging

### Oxidative stress

**Fig. 2:**
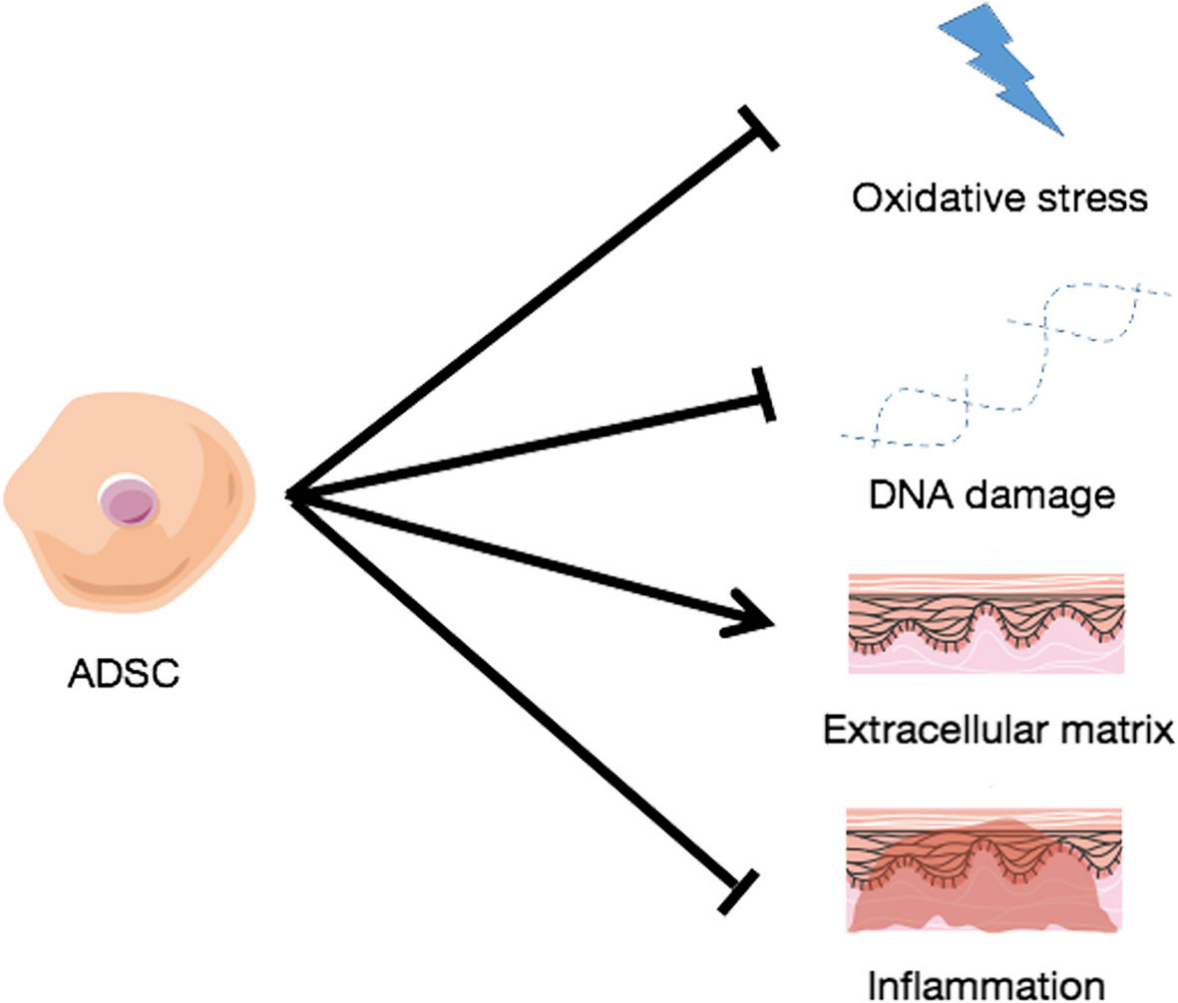
Schematic representation of the effects of ADSCs on photoaging

Oxidative stress is a major cause of photoaging [62]. It is defined as the imbalance between ROS and antioxidants. Some studies support the protective function of ADSCs and secretome of ADSCs during oxidative injury (Fig. 2). For example, HGF (hepatocyte growth factor) is reported to protect the retinal pigment epithelium [63], the heart [64], and the liver [65] against oxidative stress. VEGF leads to great decrease of renal ischemia-reperfusion (I/R)-induced oxidative stress in mice [66]. IL-6 attenuates oxidative stress by activating the downstream signal transducer and activator of transcription 3 (STAT3), nuclear factor erythroid 2-related factor 2 (Nrf2)-antioxidant pathway and upregulating manganese superoxide dismutase (Mn-SOD) [67, 68]. However, TNF- *α* can induce ROS generation in retinal pigment epithelial [69]. It may be induced by proinflammatory effect of TNF- *α*. In addition, it is reported that conditioned medium from ADSCs (ADSC-CM) and exosome of ADSCs(ADSC-Exo) protect alveolar epithelial cells [70], keratinocytes [71], human dermal fibroblasts (HDF)s [71–73], dermal papilla cells [19] against oxidative stress. Specifically, the antioxidant capacity of the ADSC-CM is 1.8 times higher than that of the standard medium [74]. Mitochondrial-derived reactive oxygen species (mtROS) is associated with inflammasome activation [75]. Elevated ROS levels will lead to increase in mitochondrial lipid peroxidation. It is reported that ADSCs are able to suppressing mtROS levels in stressed recipient cells [76]. However, glycoprotein A repetitions predominant (GARP) silencing in ADSCs increased their activation of transforming growth factor- *β* (TGF- *β*) that augmented the levels of mtROS [30].

ADSCs fight against oxidative stress through higher antioxidant enzymes expression such as GPx [72], SOD [72, 77], catalase [78]. They also upregulate antioxidant response element such as phase II gene HO-1 [71] and suppress the production of myeloperoxidase (MPO) [79] that can induce lipid peroxidation, reactive chlorinating and brominating species, NADPH oxidase (NOX)1 and 4 [80], and malondialdehyde (MDA), the most commonly used marker for lipid peroxidation [77, 81, 82]. ADSCs depend on Nrf2 to downregulate NOX1 and NOX4 and upregulate HO-1 [80]. In wound beds, overexpressed Nrf2 of ADSC-Exo promoted granulation tissue formation, angiogenesis, increased the expression of growth factor, and decreased proteins related to inflammation and oxidation [31]. However, the specific mechanism of how ADSCs react on these enzymes and the precise pathway is yet to be determined.

### DNA damage

One of the major types of oxidative DNA damage products induced by free radicals is 8-hydrox-2 ^′^-deoxyguanosine (8-OHdG), and ADSCs can significantly suppress the 8-OHdG levels in the rat model [83]. Attenuated level of oxidative stress definitely contribute to reducing DNA damage. Besides, ADSCs can downregulate the expression of phosphorylated histone family 2A variant (*γ*H2AX) protein, which responses to DNA double strand breaks in irradiated cells [84]. Besides, ADSCs with overexpressed hypoxia-inducible factor (HIF)1 *α* can decrease oxidative stress and subsequent DNA damage efficiently [32]. Reduced DNA damage level can ameliorate oxidative stress in turn and exert protective capacity.

It is generally accepted that UV-induced apoptosis is generated by UV-mediated DNA damage [85]. Therefore, decrease of DNA damage has an important impact on apoptosis inhibition. Studies showed that ADSC-CM inhibited the apoptotic cell death induced by UVB and it is illustrated by the reduced sub-G1 phase of HDF [86]. Furthermore, ADSCs inhibit apoptosis by transporting and regulating proteins. For example, ADSCs-Exo not only remarkably reduced hypoxia and serum deprivation (H/SD)-induced apoptosis in murine long bone osteocyte (MLO)-Y4 cells [87], but also promoted cell proliferation and migration of human keratinocytes (HaCaT) cells, and decreased cell apoptosis of HaCaT cells, both via upregulating the radio of B cell lymphoma-2 (Bcl-2)/Bcl-2 associated X(Bax) [88]. In a skin flap transplantation model, H_2_O_2_-treated ADSC-Exo group had fewer apoptosis cells, resulting in higher mean percentage of skin flap survival area after I/R injury [33]. In the mean time, apoptotic biomarkers (Bax/caspase-3/poly ADP-ribose polymerase) were significantly reduced through the combination of ADSC-Exo and ADSCs in another acute kidney I/R injury model [89]. In addition, hypoxia-treated ADSCs downregulated the expression of pro-apoptotic gene such as CASP9, BAX, BID, and BLK and upregulated the expression of anti-apoptotic gene BCL-2 in hepatocytes [90]. Last but not the least, it is reported that ADSCs can convert necrotic or late apoptotic cells to early apoptotic cells in photoaged fibroblasts depending on paracrine capability [22].

Senescence is a state of stable cell growth arrest in the G1-phase [91]. The major inducer of the cell cycle arrest is p21, which is downregulated by ADSCs in fibroblasts [34]. ADSC-CM treatment decreased cellular senescence induced by UVB and SA- *β*-Gal in HDFs [92], which can be explained by the reduced oxidative stress and attenuated DNA damage based on the oxidative stress theory. Besides, overexpression of VEGF in ADSCs promoted the function of ADSCs on downregulating SA- *β*-Gal and inhibiting senescence in fibroblasts injured by UVR [34]. It is reported that p53 were significantly downregulated in hematopoietic stem cells (HSCs) cultured on ADSCs, which may contribute to the capacity of ADSCs for inhibiting apoptosis and senescence [93].

### Extracellular matrix

Collagen is a primary element in ECM. When exposed to UVR, it will be degraded mostly because of increased activity of MMPs caused by ROS production [71]. The molecular interaction between fibroblasts and the ECM is obstructed due to the decrease of collagen, which eventually results in the damage of fibroblast function and further collagen decrease [94]. It is reported that ADSC-Exo can promote the migration ability of HDFs irradiated by UVB [95], suppress the overexpression of MMP-1 [95, 96], MMP-2 [95, 97], MMP-3 [95, 96], MMP-9 [92, 95] and MMP-13 [97] caused by UVB. Moreover, it can increase collagen I, II, III, and V and elastin expression [92, 95]. Inhibitor of metalloproteinase (TIMP)-1 and TGF- *β*1 that are critical factors contributing to suppressing MMP and synthesizing ECM were upregulated in extracellular vesicles from ADSCs (ADSC-EV)-treated HDFs after UVB exposure [95]. Injection of ADSCs in nude mice promoted collagen density, fibroblast number, and skin thickness [35]. Besides, procollagen type I protein that accounts for the synthesis of dermal collagen increased noticeably [98].

Elastic fibers accumulate abnormally in sun-aged skin. Subcutaneous injection of ADSCs significantly decreased elastosis and resulted in new oxytalan elastic fiber production in the papillary dermis. It also promoted the tridimensional architecture in the reticular dermis and a richer microvascular bed structure [99], concomitant with activation of cathepsin K and matrix MMP 12 [100].

Hyaluronic acid (HA) is one type of GAGs, which decreases in photoaged skin [101]. HA composes proteoglycan (PG) aggregates that are large compound of HA and PGs bound to HA. Their combination with other matrix proteins, like collagen networks, leads to the formation of supermolecular structures and increases the hardness of the tissue [102]. HA production and degradation modulation are important for ECM homeostasis maintenance. ADSCs produce TGF- *β*1, basic fibroblast growth factor (bFGF), epidermal growth factor (EGF), and platelet-derived growth factor (PDGF)-BB, which promote the expression of HA synthase in fibroblast [103].

ECM is secreted mainly by fibroblasts. As already mentioned, ADSC-CM reduced cellular senescence and apoptosis in irradiated HDFs. And it is demonstrated that ADSC-CM plays a protective role in preventing HDFs from extrinsic aging damages. Among them, PDGF-AA may promote the function of ADSC-CM with some other elements [92]. Moreover, in in vitro and in vivo experiments, the Wnt/ *β*-catenin signaling pathway can promote the activation of skin fibroblasts through the transplantation of ADSCs. The combination of ADSCs and fractional CO_2_ laser can further strengthen the dermal fibroblast activity [21]. ADSC-Exos can be absorbed by fibroblasts and enhance their ability to proliferate and migrate, as well as promote the deposition of collagen type I and III through the PI3K/Akt signaling pathway [104]. ADSC-CM is currently under clinical study to treat skin aging due to its ability to promote fibroblast migration and collagen synthesis [105].

It is also found that ADSC-CM can suppress the activation of the MAPK [71] and ERK1 [73] signaling pathway induced by UVB, promote TGF- *β*, suppress IL-6, and inhibit activation of AP-1 and NF- *κ*B [71] signaling molecules induced by UVB. They all showed that in the early UVB responsive stage, ADSC-CM can regulate relative molecules to exert its function.

Some research suggested that UVB causes photoaging by changing skin stem cell niches that are mainly composed of ECM and other regulatory factors [106]. Transferred ADSCs can influence the BMP signaling pathway and differentiate into skin stem cells to remodel the niches, as a supplement to the paracrine mechanism of the protective function of ADSCs.

### Inflammatory response

Attenuated oxidative stress is concomitant with reduced inflammation because oxidative stress occurs after UVR results in glucocorticoid resistance and the subsequent progress of skin inflammation [107]. What is more, accumulation of senescent cells causes the increase of proinflammatory molecules such as IL-6, IL-8, and TNF- *α* that are closely related to chronic inflammation [24]. ADSC can reduce senescent cells and may alleviate the inflammatory responses in this way.

Besides, ADSCs can mediate inflammatory response directly. ADSCs are able to ameliorate inflammatory and immune responses [108], which is illustrated in several types of cells, such as natural killer T cells [109], regulatory T cells [110], T cells [111], and dendritic cells [112]. In the injured area, ADSCs were aggregated and relocate by increasing CXCR-4 expression. What is more, in this way, the inflammatory phenotype of immune cells will be transferred into anti-inflammatory cells [24]. In vitro, ADSC-Exo had an inhibitory effect on the differentiation of CD4+ or CD8+ T cells toward effector or memory cell phenotypes, regulated by anti-CD3/CD2/ CD28 stimulation[113]. Moreover, ADSCs increased macrophage recruitment and promoted macrophage polarization toward anti-inflammatory M2 phenotypes by secreting TGF- *β*, IL-1 *β*, and IL-6 [114, 115]. In activated macrophages, the generation of pro-inflammatory cytokines like TNF- *α* and IL-12 and the trend of apoptosis can be suppressed by ADSCs [116]. What is more, regulatory T cells’ differentiation and proliferation are suppressed by ADSCs while regulatory T cells’ are enhanced. It seems that the anti-inflammatory function of ADSCs is associated with the phenotypic differentiation of T cells [116].

In fact, ADSC secretome includes various proinflammatory and anti-inflammatory components including growth differentiation factor (GDF)11, TGF- *β*, bFGF, VEGF, toll-like receptor (TLR)2, TLR4, IL-10, and MMP [117–122]. The final outcome is determined by the balance of these anti-inflammatory and pro-inflammatory molecules. In pig models with both I/R and hemihepatectomy, ADSCs transplantation successfully improved high concentrations of pro-inflammatory cytokines such as IL-1 *β*, IL-6, and TNF- *α* induced histopathological injury [123]. Moreover, ADSCs promoted the expression of IL-10, regenerative molecules like HGF, Cyclin D1, proliferatory molecules such as VEGF, angiotensin (ANG)-1, and ANG-2 [123]. Recently, microRNA (miR)-146a-transfected ADSCs and the secretome containing abundant miR greatly demonstrated their angiogenic and anti-inflammatory abilities [36].

Furthermore, it is reported that ADSCs can modulate the UVB-induced inflammatory signaling pathways. UVB improved the inflammatory molecules expression such as phospho-NF- *κ*B p65, nod-like receptor protein (Nlrp)3, vascular cell adhesion molecule (VCAM)-1, COX2, and TNF- *α*, while ADSCs transplantation repressed the overexpression of these genes [97].

Inflammation and apoptosis are considered to be the main features of skin photoaging [124, 125], while ADSCs can suppress this inflammatory progress. These findings about ADSCs could provide effective strategies to deal with photoaging.

## Potential applications of ADSCs and ADSC secretome in photoaging and photocarcinogenesis

Recent clinical research suggested that ADSCs can be applied to ameliorate multiple skin conditions, because they can stimulate injured skin to regenerate [126]. The efficacy of ADSCs under multiple skin aging conditions have been presented and confirmed and are significant in potential therapeutic applications development, such as anti-wrinkling, dermal thickness improvement, skin whitening, UV-induced skin injury regulation, and tumor applications. A randomized controlled trial study showed that protein extracts of ADSC-CM via microneedles presented a critical improvement for melanin levels, brightness, skin gloss, roughness, elasticity, and wrinkles without the unfavorable side of the skin. Besides, more than 70% of the participants of the participants said that in the test surface, wrinkles, firmness, elasticity, hydration, whitening, and brilliance were noticeably improved [127].

Next, the therapeutic potential as well as the current limitations of ADSCs in photoaging and photocarcinogenesis will be described in multiple aspects. The pre-clinical studies of ADSCs and their secretome in photoaging and photocarcinogenesis are listed in Table 2.

**Table 2:** Pre-clinical studies of ADSCs and their secretome in photoaging and photocarcinogenesis

Function	In vitro or in vivo	Source	Effect cell	Findings	Reference
Anti-wrinkling	In vitro	ADSC	HDF	Stimulate collagen expression with higher tropoelastin and fibrillin-1 assessments, decrease MMP expression	[20]
Anti-wrinkling	In vitro and in vivo	ADSC and ADSC-CM	HDF	Augment dermal thickness and stimulate the proliferation of HDF by the Wnt/ *β*-catenin signaling pathway	[21]
Anti-wrinkling	In vitro	ADSC-CM	HDF and HaCaT	Increase procollagen type I synthesis inhibitors IL-6, promote collagen synthesis enhancer TGF- *β* and inhibit UVB-induced activation of AP-1 and NF- *κ*B	[71]
Anti-wrinkling	In vitro and in vivo	ADSC and ADSC-CM	HDF	Reduce wrinkle, decrease the UVB-induced apoptotic cell death and MMP1 expression, increase collagen I	[86]
Anti-wrinkling	In vitro	ADSC-EV	HDF	Suppress the overexpression of MMP-1, -2, -3 and -9 and enhance the expression of TIMP-1, TGF- *β*1, collagen types I, II, III and V and elastin	[95]
Anti-wrinkling	In vivo	ADSC	HDF	Stimulate collagen synthesis in HDF and increase angiogenesis	[98]
Whitening	In vivo	ADSC	Melanocyte	Inhibit melanin formation	[128]
Whitening	In vitro	ADSC-CM	Melanoma B16 cell	Inhibit melanin synthesis by downregulating tyrosinase and TRP1, mainly mediated by TGF- *β*1	[129]
Whitening	In vivo	ADSC	Melanocyte	Attenuate tanning following UVB-irradiation by suppressing tyrosinase activity	[130]
Wound healing	In vivo	ADSC	HDF	Inhibit senescence and recover from the injury caused by UV by downregulating SA- *β*-Gal, p21 and MMP-1	[34]
Wound healing	In vitro	ADSC-Exo	HDF	Enhance proliferation and migration of HDF, as well as promote collagen type I and III deposition via the PI3K/Akt signaling pathway	[104]
Inhibit skin cancer	In vivo	ADSC	Total cell of the skin	Restore skin barrier by ameliorating the downregulation of *α*6 integrin, CD34, and collagen I by UVB, reducing the overexpression of COX2 and TNF- *α* induced by UVB.	[97]
Inhibit skin cancer	In vitro and in vivo	ADSC-CM	Melanoma B16 cell	Decrease the proliferation and migration ability of B16 melanoma cells and reduce volume of the tumor mass	[129]
Systematic improvement	In vivo	Protein extracts of ADSC-CM	Total cell of the skin	Improve melanin levels, brightness, skin gloss, roughness, elasticity, and wrinkles	[127]

### Anti-wrinkling and skin thickness improvement

Although the mechanism of wrinkle formation is not well understood, there is general atrophy of the ECM, fewer fibroblasts, and reduced synthetic ability [131, 132]. The generation of wrinkles is modulated by genetic factors, but the amount of exposure to UVR is also a significant component. UVR increases the degradation of collagen and elastic fibers, thereby leading to photoaging through the wrinkle formation and the skin elasticity loss [133].

Photoaging is a complicated process that is similar to dermal wounds pathologically [26]. Dermal fibroblasts communicate with keratinocytes, adipose cells, and mast cells, exerting essential functions in these progress. Meanwhile, they also synthesize ECM proteins, glycoproteins, adhesive molecules, and various cytokines [134]. Dermal fibroblasts play an important role in the fibroblast-keratinocyte-endothelium complex by providing these factors and promoting interactions between cells, which promotes wound repair as well as keeps the dermal integrity and skin youth. Normal applications dealing with dermal aging like laser and topical regimens usually promote the synthesis of ECM through activation of fibroblast. It was reported that ADSCs activated HDF through the generation of various growth factors which promote the proliferation and relocation of HDF and regulate the secretion of collagen in HDF [86]. It was also reported that ADSCs have anti-wrinkle functions in animal models based on the capacity of activating fibroblast. For example, ADSCs augmented skin thickness and stimulated the proliferation of dermal fibroblast on the photoaged skin by the Wnt/ *β*-catenin signaling pathway in both vitro and vivo experiment [21]. In an experimental study using mice, treated with ADSC-CM, UVB-induced wrinkles in nude mice were noticeably ameliorated, which is mainly regulated by the decrease of apoptosis induced by UVB and increase of collagen synthesis in HDF [86]. Collagen synthesis by fibroblasts is promoted by multiple factors including insulin-like growth factor (IGF), EGF, IL-1, and TNF- *α*, but TGF- *β* seems to be the most important stimulator in vivo [135, 136].

In a comparative study, both ADSC group and fibroblast group showed decreased wrinkle area. Compared with ADSCs, fibroblasts promoted more collagen expression, but they also augmented the expression of MMPs, while ADSCs reduced MMP expression [20]. ADSCs also stimulated higher collagen density and had high levels of tropoelastin and fibrillin-1 than fibroblast group, which indicates the superior regeneration capacity of ADSC.

Besides, in an athymic mouse model of photoaging, injection of ADSCs combined with HA gel ablated photoinduced skin wrinkles [37]. In a porcine acute wound model, ADSCs seeded onto collagen scaffolds increased dermal thickness and increased ECM in comparison to scaffold only and unprocessed porcine skin [38]. Moreover, the wrinkles in the areas injected with the stromal vascular fraction (SVF)/ADSC-concentrated nanofats were significantly attenuated and photoaged condition of hairless mice skin was greatly improved [35]. In addition, ADSCs and fat graft have a wrinkle-reducing effect in aged mice by synergistically affecting collagen synthesis and neovascularization [39]. Combination of ADSCs with other material or graft offers the skin a consistent and stable volume fill, which tends to augment skin thickness and reduce wrinkles more significantly.

What is more, low-level laser (LLL) preconditioning enhanced ADSCs proliferation and increased their growth factors generation and the dermal thickness of photoaged mouse skin[40], which indicated LLL might improve the clinical therapeutic potential of ADSCs. Nevertheless, the key point should be highly considered is that therapies based on stem cells still have some concerns related to safety and immune rejection [137]. Several days after transplantation, stem cells are likely to undergo apoptosis [138]. However, their secretome contains a variety of bioactive factors, such as cytokines, growth factors, and chemokines. They can function as paracrine tools and are more potential than cell transplantation. Therefore, more cell-free studies associated with ADSC-CM or ADSC-Exo can be done to fill the void.

### Skin whitening

Most of UVR stress can be defended by melanin pigmentation, but threatening health and esthetic problems will be induced by abnormal pigmentation like melasma, freckles, and senile lentigines [133]. One of the most common skin disorders is hyperpigmentation that affects all ethnic groups, mainly caused by UV exposure and skin inflammation [139]. Upon exposure of the skin to UVR, melanogenesis is facilitated by the stimulation of tyrosinase. Tyrosinase is a rate-limiting enzyme in the melanin biosynthesis cascade, which catalyzes the hydroxylation of tyrosine to L-3,4-dihydroxyphenylalanine (L-DOPA) and the subsequent oxidation of L-DOPA to dopaquinone [140]. In the absence of thiols, dopaquinone can undergo cyclization into dopachrome and turn to dihydroxyindole-2-carboxylic acid-melanin. By the activation of tyrosinase-related proteins 1 and 2 (TYRP1, TYRP2), the dark brown-black insoluble type of melanin finally forms [141].

Conventional methods dealing with hyperpigmentation such as acid peels and topically used hydroquinone creams can cause acute contact dermatitis or skin pigment spots. Besides, chemical peels do not work on deep wrinkles or pigmentations [142]. However, ADSCs are more potential to treat aged skin by stimulating skin regeneration ability and inhibiting melanin, as well as overcoming current clinical limitations. It is reported that ADSCs can exert as whitening agents via a paracrine function or their own presence [128, 129]. For example, ADSCs can secrete various factors, mainly TGF- *β*1, to exert their whitening effect in vitro. The tyrosinase and TRP1 expression are downregulated by TGF- *β*1 in B16 melanoma cells [129]. However, the accurate mechanism and molecular pathway about how TGF- *β*1 regulates melanin synthesis are still not quite explicit. Other cytokines can also suppress pigmentation by interacting with tyrosinase. Nevertheless, the concentrations of these components in ADSC-CM are far from reaching the half-maximal inhibitory concentration (IC50) value of inhibition [72, 129]. Moreover, the skin tanning induced by UVB in mice can be ameliorated by subcutaneous ADSCs injection, which is effected by suppression of tyrosinase activity and DOPA-positive melanocytes [130]. What is more, it is reported that antioxidants suppress the formation of melanin and the transfer of melanosome as well as alter the melanin type [129, 143]. And it has already been demonstrated above that ADSC-CM has great antioxidant capacity. In a word, ADSCs could exert whitening functions as antioxidants and TGF- *β*1 also plays an important role.

### UV-induced skin injury regulation

Overexposure to UVR leads to acute skin injury that triggers the relocating of the activated immune cells into the injured skin site [144]. It forms a localized inflammatory environment and further exacerbates inflammation, resulting in delayed wound healing [145]. What is more, in C57BL/6 mice, UVR can deteriorate skin wound healing [146]. The cytokine and growth factor secreted by ADSCs function importantly in all three phases of wound healing. The first phase engages TGF- *β*, TNF- *α*, PDGF, IL-1, and IL-6, which balance the progress of inflammation [147]. In the second stage, FGFs, TGF- *β*, PDGF, HGF, IGF-1, and EGF, together with IL6, IL8, and TNF- *α*, play a critical role [148–154]. In the third phase, TGF- *β*, TNF- *α*, EGF, and IL-1 contribute to remodeling the injured site. [155–160]. Besides, ADSCs are able to migrate to injured sites effectively and boost adjacent cell to regenerate. Moreover, ADSCs have been shown to differentiate into lots of skin cells such as keratinocytes and dermal fibroblasts (DF) [161–163].

In a UV-injured mice model, skin appearance of ADSCs group was noticeably improved with less wrinkles, pigmentation, or erythema and the skin recovered from the injury caused by UVR better [34]. As we all know, VEGF exerts angiogenesis function by interacting with vascular endothelial cells and promoting blood vessels proliferation. Overexpression of VEGF in ADSCs further allowed the skin to resist photoaging. The VEGF group almost recovered from the UVR-induced injury and their skin conditions looked like those in the negative control group [34].

There were already plenty of studies of ADSCs in wound healing applications [164, 165], but only a few reports on the UV-induced skin injury. Therefore, more studies could be done to investigate the potential application of ADSC in UV-induced skin injury.

### Skin cancer inhibition

Photoaging and skin cancer are triggered mainly by UVR from chronic sun exposure. And they share lots of mutual molecular and histological changes. For instance, mutation in p53 gene, an essential factor in photoaging model, has also been recognized as a significant biomarker in skin cancer induced by UVR [166] and been found in different skin cancers, especially squamous cell carcinomas [167] and basal cell carcinoma [168]. Besides, ROS contributes to the progress of skin cancer, while its exact role in skin cancer has not been thoroughly elucidated [169]. Moreover, inflammatory responses induced by photoaging could be caused by photocarcinogenesis and degradation in ECM could be responsible for tumor dissemination [170], while ADSCs can ameliorate the inflammatory response and inhibit the degradation in ECM. These results might offer us potential methods to inhibit photoaging and photocarcinogenesis.

Multiple studies are interested in the interaction of ADSCs and the oncogenic process. Indeed, mesenchymal stem cells (MSCs) can regulate cancer indirectly or exert a direct function by transforming malignantly [171]. Contradictory results have been presented that ADSCs can exert pro-tumor or anti-tumor function, both in vitro and in vivo. For example, in a mouse model of xenotransplantation of human breast cancer, the study presented that ADSCs injected into a tumor can promote tumor growth. However, when injected around the tumor, ADSCs inhibited tumor growth, suggesting that distinct influences the effect of ADSCs in different tumor microenvironments [172]. A study in vitro showed that ADSC-EV promoted Wnt/ *β*-catenin signaling to facilitate MCF7 human breast carcinoma cell proliferating and migrating [172], although effects of angiogenesis were not assessed. Another in vitro study illustrated that ADSCs-CM can slow down liver cancer cells growth through suppressing cell proliferation and increasing cell apoptosis, as well as inhibiting cell motility, adhesive ability, migration, and invasion [173]. Besides, it is also reported that human glioblastoma cancer stem cell subpopulations are not affected by ADSC-CM [174]. Therefore, before drawing conclusions, further detailed research in this area is needed.

As for skin cancer, a study illustrated that ADSC-CM greatly suppressed the migration capacity of B16 melanoma cells and reduced the volume of the tumor mass [175]. ADSCs can restore skin barrier by ameliorating the downregulation of *α*6 integrin, CD34, and collagen I by UVB, reducing the overexpression of COX2 and TNF- *α* induced by UVB [97]. ADSCs engineered to express interferon (IFN)- *β* and combined with cisplatin can migrate to the tumor sites and inhibit the growth of melanoma more effectively, as well as led to extended survival time. It suggested that ADSC can be used as a powerful cell-based delivery vehicle to release therapeutic drugs to tumor lesions [41]. Besides, it is already illustrated above that ADSCs can suppress photoaging- and photocarcinogenesis-related inflammatory responses and ECM degradation [97]. Collectively, ADSCs and their secretome are quite potential as a therapeutic anti-skin cancer medicine or a delivery vehicle. However, more basic research and clinical trial must be conducted to find out molecular mechanism details.

## Conclusion and perspective

Photoaging is a complex process triggered mainly by UVR from chronic sun exposure. This leads to DNA damage and ROS production, which initiates an inflammatory response altering cell structure and function. The harmful effects of oxidative stress exert through a variety of mechanisms, which involve changes in proteins and lipids, induction of inflammation, immune suppression, DNA damage, and activation of signal transduction pathways that affect gene transcription, cell cycle, and proliferation. And it finally leads to cell death, apoptosis and senescence, degradation of dermal collagen, and degeneration of elastic fibers as well as chronic inflammation and skin cancer.

In regard to photoaging, the treating strategies aim to ensure patient satisfaction in fields of esthetic appearance and functionality. ADSCs can reduce oxidative stress, inhibit cell apoptosis and senescence, improve ECM synthesis and skin regeneration, and regulate the inflammation progress. Besides, studies have demonstrated that ADSCs may have multiple clinical therapeutic applications, such as tissue regeneration, anti-wrinkle, tumors, and depigmentation. ADSCs are thought to be “immuno-privileged” and reliable in culturing for a long time [176], thereby exerting an outstanding advantage in dermatological field. In conclusion, these promising consequences showed that ADSCs might be potential cosmo-therapeutic tools addressing photoaging problem.

However, there are many problems with the applications of ADSC in dermatology, such as lack of details on how ADSC affects keratinocytes, fibroblasts, and endothelial cells or acts as a carrier for the secretion of soluble factors. [1]. Additionally, systemic and local delivery may have effects in multiple cell types simultaneously, sometimes with opposing outcomes. Thus, possible side effects should be taken into account and the safe doses should be determined personally. Moreover, the application of ADSCs could be impaired by some limitations. It is reported that the formation of tumor can be induced by transportation of MSCs into normal tissues [177]. ADSC-CM was especially known for high level of factors involved in cancer progression and may have unexpected side effects. Recent studies have also demonstrated that ADSCs after transplantation do not survive for a very long time. Besides, Pap cervical smear used for obtaining human uterine cervical stem cells (hUCESCs) is less invasive and less painful than liposuction used for obtaining ADSCs [178].

In terms of manufacturing, storage, handling, and safety, secretome-based approaches using conditioned medium or exosomes may bring huge potential benefits than living cells [178]. We can expect that cell-free secretomes rather than ADSCs are more potential in treatment of skin aging. What is more, inducing secretory modifications in ADSCs are promising to overcome the current limitations and enhance the anti-photoaging capacity.

Further study about the molecular details regarding the involvement of ADSCs in photoaging applications need to be carried out in order to increase our understanding and open the way to therapeutic approaches. Besides, in order to establish the optimal, durable, and safe strategy for ADSCs and ADSC secretome in the treatment of patients with symptoms of photoaging and aging, long-term and extensive in vivo studies are absolutely necessary.

## Abbreviations

8-OHdG: 8-Hydrox-2’-deoxyguanosine
ADSC: Adipose-derived stem cell
ADSC-CM: Conditioned medium from ADSC
ADSC-EV: Extracellular vesicles from ADSC
ADSC-Exo: Exosome of ADSC
ANG: Angiotensin
AP-1: Activator protein 1
Bax: BCL-2 associated X
Bcl-2: B cell lymphoma-2
bFGF: Basic fibroblast growth factor
CXCR: C-X-C chemokine receptor
DF: Dermal fibroblasts
ECM: Extracellular matrix
EGF: Epidermal growth factor
ERK: Extracellular regulated protein kinases
GAG: Glycosaminoglycan
GARP: glycoprotein A repetitions predominant
GDF: Growth differentiation factor
GPx: Glutathione peroxidase
H/SD: Hypoxia and serum deprivation
HA: Hyaluronic acid
HaCat: Human keratinocytes
HDF: Human dermal fibroblast
HGF: Hepatocyte growth factor
HIF: Hypoxia-inducible factor
HO-1: Heme oxygenase-1
HSC: Hematopoietic stem cell
hUCESC: Human uterine cervical stem cell
I/R: Ischemia-reperfusion
IC50: Half-maximal inhibitory concentration
IFN: Interferon
IGF: Insulin-like growth factor
IL: Interleukin
L-DOPA: L-3,4-dihydroxyphenylalanine
LLL: Low-level laser
MAPK: Mitogen-activated protein kinase
MDA: Malondialdehyde
MiR: microRNA
MLO: Murine long bone osteocyte
MMP: Matrix metalloprotease
Mn-SOD: Manganese superoxide dismutase
MPO: Myeloperoxidase
MSC: Mesenchymal stem cell
mtROS: Mitochondrial-derived reactive oxygen species
NF- *κ*B: Nuclear factor-kappaB
Nlrp: Nod-like receptor protein
NOX: NADPH oxidase
Nrf2: Nuclear factor erythroid 2-related factor 2
PDGF: Platelet-derived growth factor
PG: Proteoglycan
ROS: Reactive oxygen species
SA- *β*- Gal: Senescence-associated *β- galactosidase*
SOD: Superoxide dismutase
STAT3: Signal transducer and activator of transcription 3
SVF: Stromal vascular fraction
TGF- *β*: Transforming growth factor- *β*
TIMP: Tissue inhibitor of metalloproteinase
TLR: Toll-like receptor
TNF- *α*: Tumor necrosis factor- *α*
TYRP: Tyrosinase-related proteins
UV: Ultraviolet
UVA: Long wavelength ultraviolet radiations
UVB: Medium wavelength ultraviolet radiations
VCAM: Vascular cell adhesion molecule
VEGF: Vascular endothelial growth factor
*γ*H2AX: Phosphorylated histone family 2A variant
